# Single-cell and Multi-omics Analysis Confirmed the Signature and Potential Targets of Cuproptosis in Colorectal Cancer

**DOI:** 10.7150/jca.104702

**Published:** 2025-01-13

**Authors:** Tao Jiang, Zijing Wang, Zhanyuan Sun, Hengyi Lv, Guo Li, Hai Li

**Affiliations:** 1 Department of Anal-Colorectal Surgery, General Hospital of Ningxia Medical University, 804 Shengli Road, Yinchuan 750004, China.; 2 First Clinical Medical College, General Hospital of Ningxia Medical University, 804 Shengli Road, Yinchuan 750004, China.; 3 Ningxia Key Laboratory of Stem Cell and Regenerative Medicine, Institute of Medical Sciences, General Hospital of Ningxia Medical University, Yinchuan 750004, Ningxia Hui Autonomous Region, China.

**Keywords:** Colorectal cancer, Cuproptosis-related genes, Immune, Single-cell, Prognosis

## Abstract

**Background:** Cuproptosis, a form of copper-mediated programmed cell death, has recently garnered significant attention. However, the mechanisms by which CRGs affect the progression of CRC remain unclear.

**Methods:** Bioinformatics approaches were employed to analyze transcriptomic datasets and clinical data from 630 CRC patients, focusing on copy number variations, prognostic implications, and immune infiltration characteristics associated with CRGs. Key CRG-related genes impacting prognosis were identified using LASSO and Cox regression methods. A prognostic model incorporating various molecular markers and clinical parameters was constructed with a training cohort and validated with a separate validation cohort. This model was used to explore clinical indicators, immune infiltration, and tumor microenvironment characteristics in CRC patients. Additionally, single-cell analysis was performed to investigate the biological roles of critical genes, and expression patterns of these genes were assessed via qRT-PCR and WB.

**Results:** A prognostic scoring model was established based on three pivotal genes associated with CRC prognosis. This model, an independent prognostic indicator, outperformed traditional clinicopathological features in predicting patient outcomes. Kaplan-Meier survival curves demonstrated superior prognostic outcomes for individuals in the low-risk group compared to those in the high-risk group. Model stability and reliability were confirmed through ROC analysis and univariate and multivariate Cox regression analyses. Further analysis revealed significant correlations between prognostic scores and the presence of M0 macrophages and memory CD4^+^ T cells. Differences in the expression of CDKN2A, PLCB4, and NXPE4 across various CRC tissues and cells were characterized using WB, IHC and qRT-PCR.

**Conclusion:** This study not only highlights the diverse omics profiles of CRGs in CRC but also introduces a novel model for accurate prognostic forecasting.

## 1. Introduction

Colorectal cancer (CRC) is a major public health challenge worldwide, ranking as the third most frequently diagnosed cancer and the second leading cause of cancer-related deaths 1, 2. While advancements in early detection and therapeutic interventions have modestly improved survival rates, the prognosis for patients with advanced-stage CRC remains poor 3. Stage IV CRC patients, for instance, have a five-year survival rate of only 10% to 15%, underscoring the urgent need for improved treatment strategies and early detection methods 4. Research highlights the importance of early screening, particularly for identifying adenomatous polyps, which are a key early indicator of CRC and are manageable if detected early 5. Despite progress in understanding CRC's molecular mechanisms, the prognosis for patients with early-onset CRC (diagnosed before age 50) has not significantly improved, prompting ongoing research into this concerning trend 6. Identifying novel CRC-specific biomarkers, investigating its pathogenesis, and discovering new targeted treatments are therefore critical.

Recent studies into cell death mechanisms have revealed the significant role of various types of programmed cell death (PCD) in cancer development and progression 7. Among these, the copper-dependent cell death pathway, known as cuproptosis, has emerged as a mechanism of particular biological relevance 8. Cuproptosis is characterized by copper ions interacting with metabolic processes within cells, particularly targeting lipoic acid components of the tricarboxylic acid (TCA) cycle 9. This interaction disrupts protein folding and destabilizes iron-sulfur cluster proteins, leading to proteotoxic stress and cell death. Furthermore, copper's dynamic modulation within the tumor microenvironment affects cancer cell proliferation, invasion, and metastasis 10. As a cofactor for growth factors and extracellular matrix enzymes such as superoxide dismutase (SOD), copper is vital for maintaining the extracellular matrix's structural integrity and supporting tumor cell growth 11. It also influences cancer cell aggressiveness by regulating matrix metalloproteinases (MMPs), which degrade the extracellular matrix and facilitate cancer cell invasion into surrounding tissues 12. Disruptions in copper balance are closely linked to various cell death modes, including apoptosis, autophagy, and ferroptosis 13. Excessive copper can increase apoptosis through oxidative stress, while copper deficiency can impair protective processes like autophagy 14. Additionally, copper plays a crucial role in shaping the tumor microenvironment by affecting inflammatory cell functions and cytokine production, thus influencing the immune landscape of tumors through mechanisms such as macrophage polarization 15. The essential roles of copper in oncogenesis and tumor microenvironment evolution suggest that targeting copper homeostasis could provide promising new anti-cancer strategies.

This research aims to identify potential molecular markers and therapeutic targets related to cuproptosis in CRC by conducting a comprehensive multi-omics analysis of cuproptosis-related genes (CRGs), including genomic, transcriptomic, and tumor microenvironmental factors. Additionally, it explores the roles of cuproptosis within the tumor microenvironment with the goal of developing innovative diagnostic, therapeutic, and prognostic strategies and models.

## 2. Methods

### 2.1 Obtaining and compiling data

*RNA* sequencing data from 701 CRC patients, including 51 normal tissue specimens and 650 tumor tissue specimens, along with their corresponding clinical and mutation profiles, were obtained from The Cancer Genome Atlas (TCGA) database (http://cancergenome.nih.gov/). Additionally, dataset GSE12945 was sourced from the Gene Expression Omnibus (GEO) database (https://www.ncbi.nlm.nih.gov/geo/). The "limma" R package was used to normalize these raw datasets, constructing expression matrices. The datasets were then merged, and batch effects were removed using the "SVA" R package. The final dataset includes various clinical characteristics of 630 CRC patients, such as age, gender, tumor type, lymph node status, metastasis, staging, survival status, and follow-up duration.

### 2.2 Screening and validation of differentially expressed CRGs in CRC

We identified 19 CRGs from the literature 16. The differential expression of these genes between normal and tumor tissues was analyzed using the Wilcoxon rank-sum test, and results were visualized with the "ggboxplot" R package. Mutation status of CRGs in CRC samples was presented using the "maftools" R package. The percentage of copy number variations in CRGs was calculated and visualized with a circos chart using the "RCircos" R package. Gene expression data from TCGA and GEO were merged, and CRG expression levels were extracted. Kaplan-Meier survival curves and prognostic network diagrams were plotted to assess the relationship between CRGs and CRC prognosis.

### 2.3 Cluster analysis of CRGs and differences between subtypes

CRC samples were classified into different subtypes based on CRG expression levels using the "ConsensusClusterPlus" R package. Survival curves were generated with the "survival" and "survminer" R packages to compare survival times among patient subtypes. Heatmaps displaying CRG expression patterns across classifications were created using the "pheatmap" R package. Principal component analysis (PCA) and differential analysis of CRG subtypes were also conducted.

### 2.4 Gene set variation analysis and single-sample gene set enrichment analysis of CRGs

Gene Set Variation Analysis (GSVA) was performed using the "GSEABase" and "GSVA" R packages to explore enrichment pathway differences among subtype gene sets, with results visualized using a heatmap from the "pheatmap" R package. Single-sample gene set enrichment analysis (ssGSEA) was employed to evaluate immune cell infiltration in tumor samples. Immune cell infiltration scores were obtained for each sample, normalized using the min-max normalization method, and merged with subtype data for statistical analysis and visualization. Box plots depicting differences in immune cell infiltration among CRG subtypes were generated using the "ggpubr" R package.

### 2.5 GO and KEGG enrichment analysis

To investigate the potential roles of differential CRGs in biological processes, molecular functions, cellular components, and specific metabolic and signal transduction pathways, Gene Ontology (GO) and Kyoto Encyclopedia of Genes and Genomes (KEGG) enrichment analyses were conducted using the "clusterProfiler", "org.Hs.eg.db", and "enrichplot" R packages 17-19. Results were visualized using bar charts and bubble charts created with the "ggplot2" R package.

### 2.6 Cluster analysis of differentially expressed genes in CRGs

Univariate Cox analysis was performed to identify the most prognostically relevant differentially expressed genes (DEGs) among CRGs (*P<*0.05). The ConsensusClusterPlus algorithm, along with partitioning around medoids (PAM) clustering methods and Euclidean distance measurement, was used to determine the optimal number of clusters 20. Differences in survival outcomes among DEG subtypes were assessed using the Chi-square test and Kaplan-Meier survival curves. Comparisons of DEGs and CRGs across different subtypes were made, and results were visualized using heatmaps and box plots.

### 2.7 Construction and validation of prognostic model

We used the "caret" R package to randomly divide the samples obtained from univariate Cox analysis into training and test sets. LASSO regression analysis was performed with the "glmnet" R package to reduce model complexity and prevent overfitting. A multivariate Cox proportional hazards model was then constructed to select genes with the most prognostic significance. A stepwise regression approach was employed to optimize the model and determine the final predictors.

Prognostic risk scores were calculated based on the final Cox model, and CRC patients were classified into high-risk and low-risk groups according to the median score. To evaluate the impact of these risk scores on survival time, we analyzed the data separately for training, test, and all sample groups. Risk curves and survival status diagrams were plotted. A log-rank test was conducted on survival time using the "survival" R package to assess the effectiveness of risk scores in prognostic prediction.

Gene expression patterns in the model were visualized using a heatmap generated by the "pheatmap" R package, displaying differences in expression among patients in different risk groups. The model's ability to distinguish between risk groups was quantified by calculating the area under the curve (AUC) of time-dependent receiver operating characteristic (ROC) curves at specific predictive time points using the "timeROC" R package. Sankey diagrams were created with the "ggalluvial" R package to visually demonstrate relationships among CRG subtypes, DEG subtypes, and patient survival status (alive or dead). Box plots, generated using the "ggpubr" R package, illustrated the relationships between CRG subtypes, DEG subtypes, and risk scores. Differences in CRG expression across risk groups were visualized with the "ggpubr", "greshape2", and "ggplot2" R packages.

### 2.8 Constructing a nomogram

A nomogram was constructed using the "survival", "rms", and "regplot" R packages to predict the survival probabilities of CRC patients based on clinical characteristics and risk scores. Calibration curves for 1-year, 3-year, and 5-year survival rates were plotted to assess the predictive accuracy of the model. Furthermore, the clinical utility of the nomogram was assessed through decision curve analysis (DCA) utilizing the “ggDCA” R package.

### 2.9 Analysis of immune cell infiltration, tumor microenvironment, and tumor mutational burden

Immune cell infiltration levels were obtained using the CIBERSORT algorithm, and samples with statistical significance (*P<*0.05) were selected. The correlation between risk scores and immune cell infiltration levels was analyzed using the Spearman correlation test, and scatter plots were created for significantly correlated cell types. Paired analyses of immune cell types with gene expression from the risk file were conducted, and heatmaps displayed the correlation strength between gene expression and immune cell infiltration levels.

To investigate the relationship between tumor microenvironment (TME) scores and patient risk groups, we merged risk grouping data with TME scores. The Wilcoxon rank-sum test was applied to evaluate differences in TME scores between risk groups, and violin plots were generated using the "ggplot2" R package.

For TMB analysis, we integrated TMB results with risk scores and gene typing data. TMB data were log-transformed to ensure normal distribution characteristics. Samples common across all datasets were filtered for consistency and comparability. Box plots comparing TMB levels across different risk groups were created using the "ggpubr" R package. Scatter plots showed the correlation between risk scores and TMB, with different gene typings marked by various colors. Additionally, waterfall plots demonstrating gene mutation patterns in tumor samples and their association with patient risk groups were generated using the "maftools" R package.

### 2.10 Analyzing microsatellite instability, stem cell correlations, and drug sensitivity

We first retrieved risk scores and microsatellite instability (MSI) status. Using the "ggplot2" and "ggpubr" R packages, we plotted percentage bar charts and box plots to illustrate the distribution and statistical differences between MSI statuses and tumor risk levels.

To explore the connection between tumor stem-like characteristics and CRC prognosis, we analyzed risk scores and *RNA* stemness scores (*RNA*ss). Scatter plots were created with the "ggplot2" and "ggpubr" R packages to visually display the relationship between these variables.

Drug sensitivity differences among risk groups were investigated through differential expression analysis and the Wilcoxon test. Box plots, generated using the "ggpubr" R package, visually presented these changes.

### 2.11 Quantitative reverse transcription-polymerase chain reaction and single-cell analysis

During surgical procedures at Ningxia Medical University General Hospital, 40 CRC patients provided tissue samples for analysis. Total *RNA* was extracted using Trizol (Takara, China) and cDNA was synthesized with Prime Script RTase (Takara, China). Quantitative real-time PCR (qRT-PCR) was performed on the Bio-Rad CFX96 System (Bio-Rad, California) with specific primers listed in Table S1.

Single-cell analysis was conducted to investigate the primary origins of gene expression for CDKN2A, PLCB4, and NXPE4 in CRC, utilizing the TISCH database (http://tisch.comp-genomics.org/home/) 21.

### 2.12 Western Blotting (WB)

Protein lysates were prepared from tissues or cell extracts by adding protease and phosphatase inhibitors to the lysis solution (Nanjing KeyGEN BioTECH Co., Ltd., China) and keeping the solution on ice for one hour 22. After centrifugation at 12,000 × g for 10 minutes at 4°C, the supernatants were collected as crude extracts. Fifty micrograms of each protein sample were separated by 10% sodium dodecyl sulfate-polyacrylamide gel electrophoresis (SDS-PAGE) using a Bio-Rad electrophoresis system, with the separation taking 1-2 hours. Proteins were then transferred to polyvinylidene fluoride (PVDF) membranes (Millipore, Burlington, MA), pre-activated with methanol for three minutes. The membranes were blocked at room temperature for one hour with Tris-buffered saline (TBS) containing 5% skim milk and then incubated overnight at 4°C with a rabbit anti-CDKN2A antibody (dilution 1:1000, Abcam). After incubation, the membranes were washed three times with 1× TBST (0.1% Tween-20 in TBS) and incubated at room temperature for two hours with horseradish peroxidase (HRP)-conjugated goat anti-rabbit IgG (dilution 1:1000, ZSGB-Bio Origene, Beijing, China) in blocking buffer. Chemiluminescent signals were detected using Enhanced Chemiluminescence (ECL) reagent (Advansta, Menlo Park, CA, USA).

### 2.13 Immunohistochemistry (IHC)

This research involved the use of formalin-fixed, paraffin-embedded (FFPE) tissue samples from both normal and tumor tissues sourced from ten patients diagnosed with colon cancer at the Pathology Department of Ningxia Medical University General Hospital. Tissue sections, each 4 µm in thickness, were prepared and underwent deparaffinization in xylene, followed by a rehydration process through a series of graded ethanol concentrations. For the purpose of antigen retrieval, sections were placed in a citrate buffer (pH 6.0) and heated in a microwave oven at 95°C for 15 minutes. After the heating process, endogenous peroxidase activity was inhibited using 3% hydrogen peroxide. The sections were then incubated overnight at 4°C with a primary antibody targeting CDKN2A. Subsequent to rinsing with PBS, the sections received a treatment with a biotinylated secondary antibody for 30 minutes at room temperature, followed by an additional 30 minutes of incubation with an avidin-biotin complex (ABC) solution. Visualization of immunostaining was achieved using a diaminobenzidine (DAB) chromogen, and the sections were counterstained with hematoxylin. The stained slides were dehydrated and mounted for microscopic analysis.

### 2.14 Statistical analysis

Data analysis was performed using R version 4.3.2 and Strawberry Perl version 5.3.1. Statistical significance was denoted by *, **, and ***, corresponding to thresholds of < 0.05, < 0.01, and < 0.001, respectively. Findings with a *P* below 0.05 were considered statistically significant, indicating a reliable degree of confidence in the results. We used the Pearson correlation coefficient for the CRGs prognostic network. For the relationship between risk scores and immune landscape factors (such as immune cell infiltration and TMB), we used the Spearman correlation coefficient.

## 3. Results

### 3.1 Differential expression analysis of CRGs

**Figure 1:**
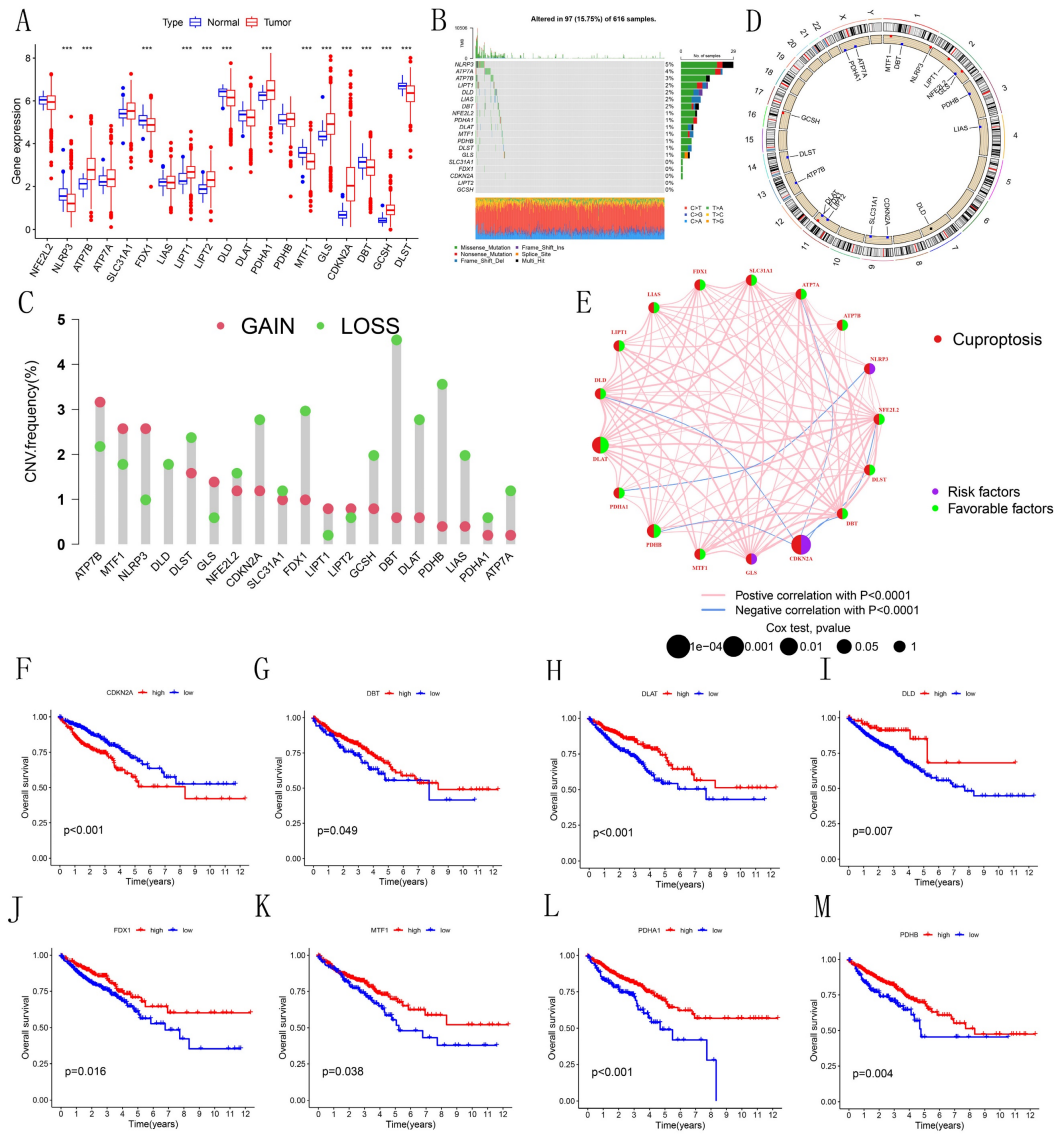
Differential Expression and Genetic Variation of 19 CRGs in CRC. (A) Box plot depicting the differential expression of CRGs across various CRC tissues. Statistical significance was assessed using the Wilcoxon rank-sum test, with differences considered significant when *P*<0.05. (B) Waterfall chart illustrating the mutation frequencies of CRGs. (C) Copy number variations (CNVs) of CRGs in 630 samples from the TCGA database. (D) Chromosomal locations of CNVs in CRGs. (E) Interaction network of CRGs. (F-M) Kaplan-Meier survival analysis of 8 CRGs associated with CRC prognosis. Symbols indicate statistical significance: *for *P*<0.05, ** for *P*<0.01, ***for *P*<0.001).

As illustrated in the boxplot (Figure 1A), 13 out of 19 CRGs demonstrated significant differential expression between normal and tumor tissues in CRC. Notably, CDKN2A, a well-known tumor suppressor gene, showed high expression in tumor tissues. The waterfall plot (Figure 1B) indicates that mutations in CRGs were present in 97 out of 616 CRC patients, with NLRP3 having the highest mutation frequency at 5%. Analysis of copy number variations (CNVs) and their circos plots (Figures 1C-D) revealed that all CRGs had a high number of CNVs. Specifically, DBT exhibited the highest frequency of CNVs, mainly due to deletions on chromosome 1, while other CRGs showed varying degrees of amplifications and deletions. These results highlight the potential significance of CNVs in shaping the genetic landscape of CRC. Intriguingly, DBT, which had the highest CNV frequency, also showed significant differential expression, suggesting that CNVs might influence the expression of genes associated with CRC. The prognostic network diagram (Figure 1E) identified GLS, NLRP3, and CDKN2A as potentially linked to poor prognosis in CRC, with CDKN2A being particularly associated with high-risk prognosis. This discovery suggests that these genes may function as significant biomarkers for the prognosis of CRC, underscoring the necessity of assessing their expression levels in clinical environments. Co-expression analysis revealed a negative correlation between CDKN2A and DLD, ATP7A, PDHB, DBT, and NFE2L2. Kaplan-Meier survival curves (Figures 1F-M) demonstrated that the expression of CDKN2A, DBT, DLAT, DLD, FDX1, MTF1, PDHA1, and PDHB was significantly associated with CRC prognosis. High expression of CDKN2A correlated with poor prognosis, whereas high expression of other genes was associated with longer survival, indicating that CDKN2A may function as an oncogene in CRC.

### 3.2 Analysis of differences between the two clusters of CRGs

**Figure 2:**
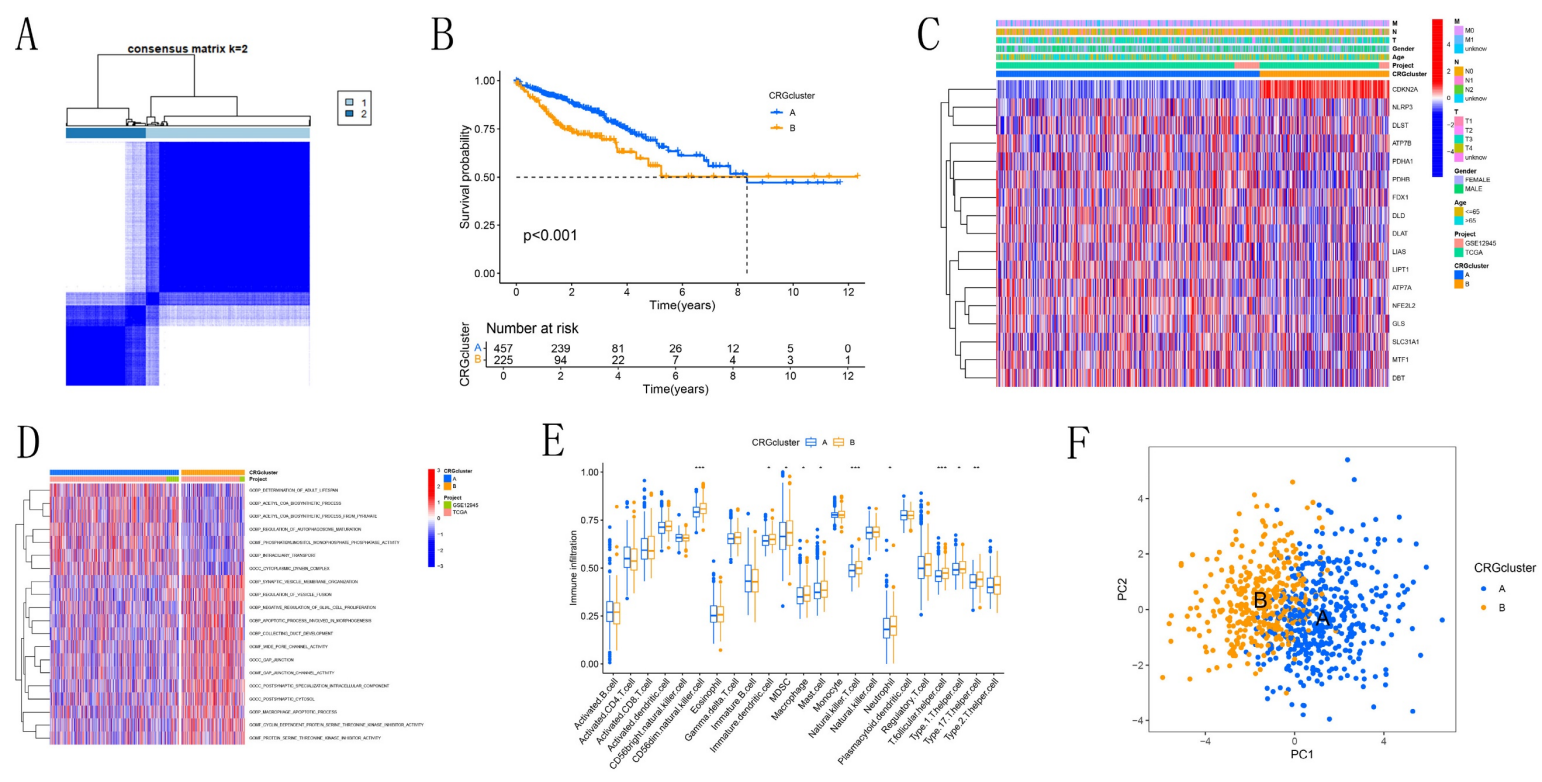
Identification of Cuproptosis Modification Patterns in CRC by Consensus Clustering. (A) Identification of two clusters through consistent clustering analysis (k = 2). (B) Kaplan-Meier survival curves comparing patients with different cuproptosis modification patterns. (C) Comparison of clinicopathological characteristics and CRG expression levels between the two cuproptosis subgroups. (D) Gene Set Variation Analysis (GSVA) of patients with different cuproptosis modification patterns to determine the activation status of biological pathways. (E) Analysis of immune cell infiltration abundance across different molecular subtypes. (F) Principal Component Analysis illustrating distinct distributions between the two subtypes.

**Figure 3:**
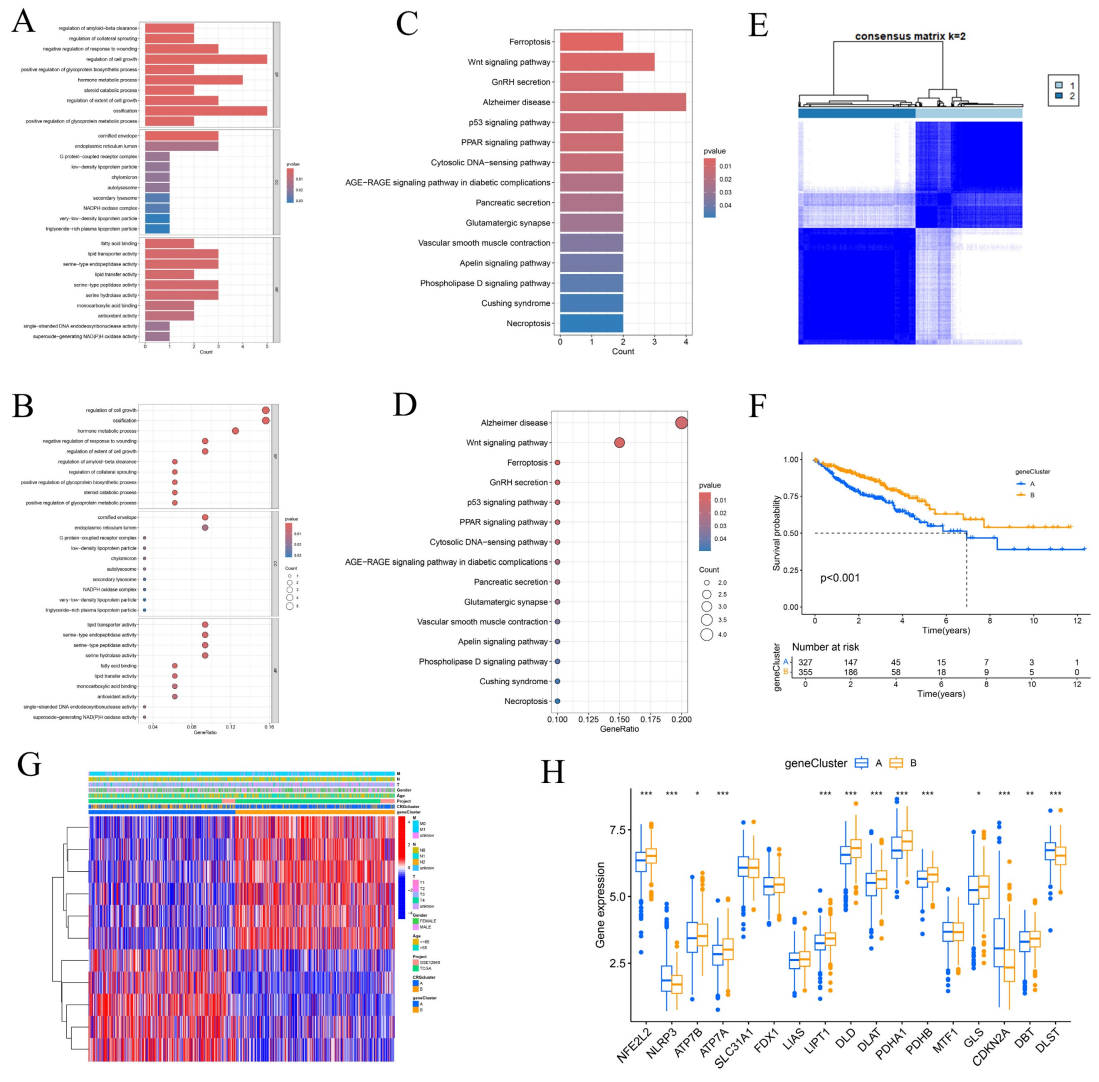
Enrichment Analysis and Identification of DEG Modification Patterns in CRC. (A-B) Bar and bubble plots illustrating the results of Gene Ontology (GO) enrichment analysis. (C-D) Bar and bubble plots displaying the results of KEGG enrichment analysis. (E) Two distinct clusters were identified through consistent clustering analysis (k = 2). (F) Kaplan-Meier survival curves for patients categorized by DEG modification patterns. (G-H) Differences in clinicopathological characteristics and expression levels of CRGs between the two DEG subgroups.

Based on the CRG expression data and clinical information of CRC patients, we determined the optimal number of clusters as K=2, dividing the samples into subtypes A and B (Figure 2A, Figure S1). Kaplan-Meier survival curves (Figure 2B) revealed that subtype A had a significantly better prognosis compared to subtype B. Heatmap analysis (Figure 2C) showed that subtype A had more favorable clinical parameters, such as TNM staging, compared to subtype B. Subtype B exhibited significant expression of CDKN2A. Subtype A was notably enriched in processes related to "ACETYL COA BIOSYNTHETIC PROCESS" and "CYTOPLASMIC DYNEIN COMPLEX," which are associated with intracellular metabolic regulation and cell signaling, as identified by GSEA (Figure 2D). Conversely, subtype B was enriched in terms related to "APOPTOTIC PROCESS," "DEPENDENT PROTEIN SERINE THREONINE KINASE INHIBITOR ACTIVITY," and "JUNCTION," which are linked to immunological modulation and cell proliferation. The activation of these processes in subtype B may indicate a tumor phenotype that is more aggressive, marked by increased cell proliferation and immune evasion. Immune cell expression, including CD56dim natural killer cells, immature dendritic cells, MDSCs, macrophages, mast cells, natural killer T cells, neutrophils, T follicular helper cells, Type 1 T helper cells, and Type 17 T helper cells, varied significantly between subtypes A and B, according to ssGSEA (Figure 2E). This indicates that variations in the immune microenvironment could play a role in the observed differences in prognosis, with subtype A likely gaining from a more advantageous immune landscape. PCA results (Figure 2F) successfully identified two distinct groups, cluster A and cluster B. This confirms the robustness of our method in distinguishing between CRC subtypes. GO and KEGG enrichment analysis (Figures 3A-D) showed significant enrichment of these CRGs in cellular processes related to cell growth and metabolism, particularly in lipid and glycoprotein metabolism, highlighting their critical role in cell cycle regulation, DNA damage repair, and energy metabolism balance.

### 3.3 Analysis of differences between the two subtypes of DEGs

Initially, 34 DEGs associated with prognosis were identified (Figure S2). Cluster analysis optimized the parameter K to 2 (Figure 3E, Figure S3), stratifying CRC patients into two distinct subtypes, A and B. Kaplan-Meier survival curves (Figure 3F) indicated that subtype B had a significantly better overall survival compared to subtype A (*P*<0.05). Clinical parameter analysis showed that subtype A was positively correlated with advanced TNM staging and reduced expression of prognosis-related genes (Figure 3G). Significant differences in gene expression were observed between the two subtypes, except for SLC31A1, FDX1, LIAS, and MTF1 (Figure 3H). Subtype A exhibited higher expression levels of NLRP3, ATP7B, CDKN2A, and DLST, reinforcing the role of CDKN2A as a carcinogenic factor. These findings highlight the distinct molecular characteristics of the subtypes and their potential implications for targeted therapy and prognosis in CRC.

### 3.4 Risk model in training and validation sets

**Figure 4:**
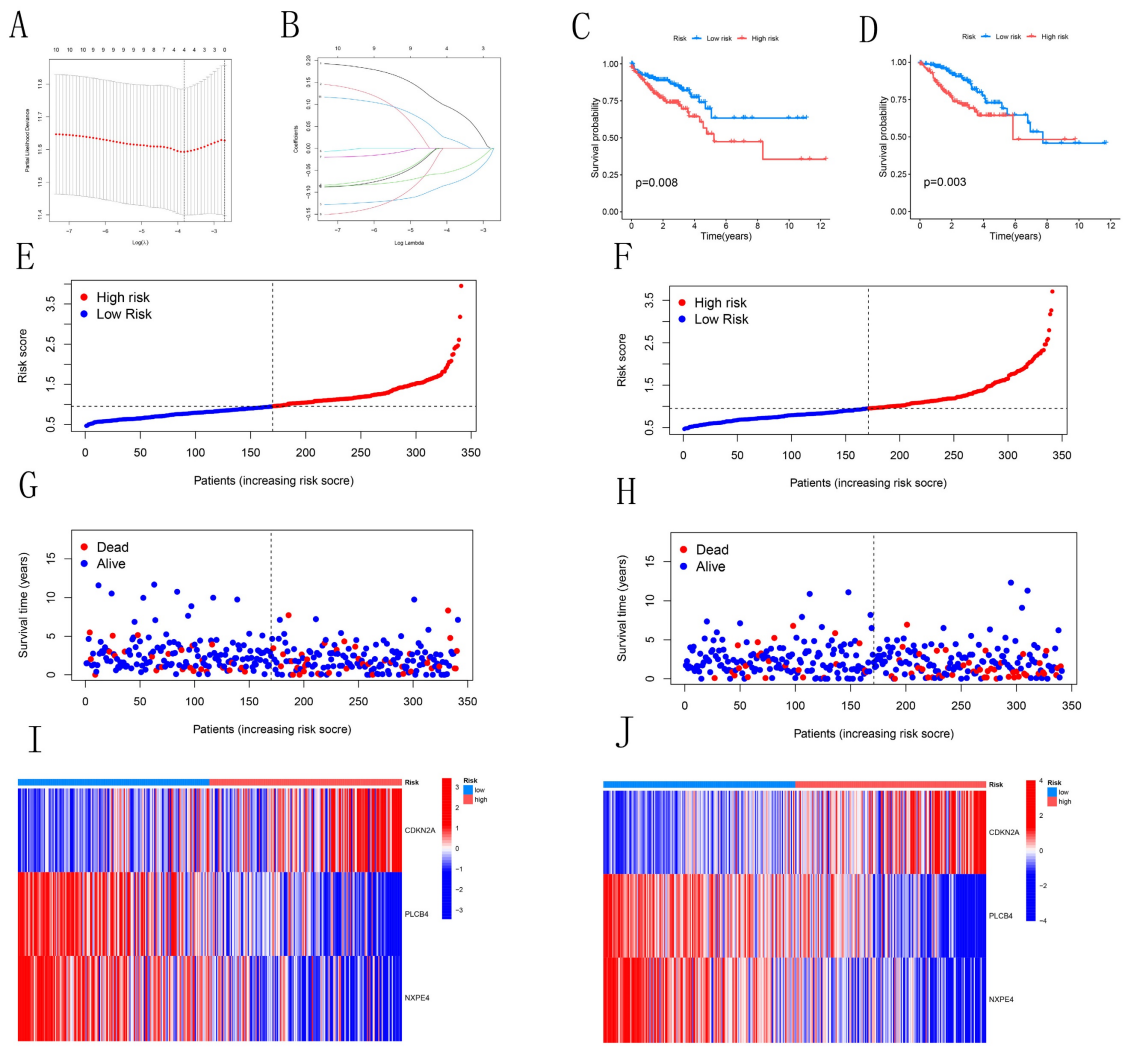
Construction and Validation of Risk Models. (A-B) LASSO variable trajectory plot and coefficient profile based on 1,000 cross-validations. (C-D) Kaplan-Meier survival curves for both the training and test sets. (E-H) Risk scores and survival status of CRC patients in the training and test sets. (I-J) Heatmaps showing the expression levels of three pivotal genes in high-risk and low-risk groups.

We identified 11 candidate genes with significant associations with CRC prognosis (*P*<0.05) through single-variable Cox analysis of 34 candidate genes. Using the LASSO model with a partial-likelihood deviation λ value of 0.0115803, we selected four genes for multivariable Cox analysis (Figures 4A-B). Ultimately, three prognostic genes (CDKN2A, PLCB4, and NXPE4) were integrated into the final multivariable Cox risk model. CRC patients were stratified into high-risk and low-risk groups based on median risk scores. The prognostic impact of this model on the overall survival (OS) of CRC patients was evaluated using Kaplan-Meier analysis. Results showed that patients in the low-risk group had a significantly higher survival rate compared to those in the high-risk group in both training and testing cohorts (Figures 4C-D). The distribution map of patient risk scores and survival status (Figures 4E-H) demonstrated a positive correlation between higher risk scores and mortality rates. Heatmaps illustrated expression differences of the three CRGs between high-risk and low-risk groups in both cohorts. Analysis of Figures 4I-J suggested similar expression patterns of various CRGs across different groups. These findings reinforce the potential of the identified risk model to guide clinical decision-making in CRC management.

### 3.5 Validation of the independent prognostic model

**Figure 5:**
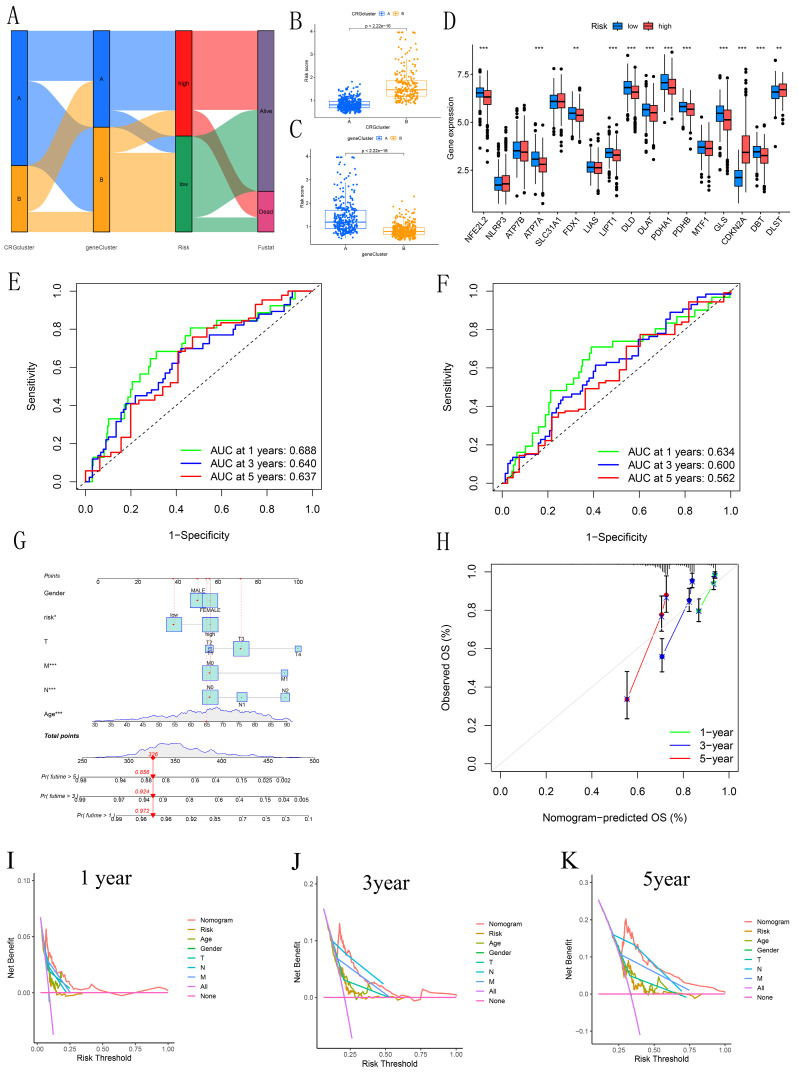
Analysis of Differences Between Clusters. (A) Sankey diagram illustrating the risk scoring and distribution between CRGs and DEGs clusters. (B-C) Box plots illustrate the risk scores and differences between CRG and DEG clusters. (D) Box plot showing the risk differences across CRGs clusters. (E-F) ROC curves estimating prognostic value. (G) Nomogram for predicting overall survival in CRC patients. (H) Calibration curves assessing the accuracy of the nomogram. (I-K) DCA to evaluate the clinical net benefit of the nomogram compared to individual factors across different risk thresholds for 1-year, 3-year, and 5-year prognosis in CRC patients.

Sankey diagrams were used to assess the distribution and correlation of patients with varying prognoses across different subtypes and risk score subgroups (Figure 5A). Figures 5B-C show differences in risk scores across various subgroups between clusters. Boxplots revealed significant differences in CRG expression between groups, with elevated expression observed in CDKN2A and DLST within the high-risk cohort, supporting the carcinogenic potential of CDKN2A (Figure 5D). ROC curves were generated to evaluate the prognostic model's performance. The AUC values for the 1, 3, and 5 years training sets were 0.688, 0.640, and 0.637, respectively (Figure 5E). Similarly, the test set showed AUC values of 0.634, 0.600, and 0.562 for 1, 3, and 5 years, respectively (Figure 5F). Furthermore, the concordance index revealed that the risk score exhibited enhanced predictive capability, consistently yielding higher values over time when compared to age, gender, and stage in forecasting patient outcomes (Figure S4). This reinforces the robustness of the risk score as a predictive tool in CRC. A nomogram was constructed for prognostic analysis, integrating various molecular and clinicopathological parameters (Figure 5G). Calibration plots highlighted the model's excellent reliability (Figure 5H). The DCA (Figures 5I-K) further illustrated the clinical utility of the nomogram. Over the intervals of 1, 3, and 5 years, the nomogram consistently provided a greater net benefit across various risk thresholds when compared to individual predictors, emphasizing its significance in clinical decision-making. Overall, the integration of these predictive models enhances the precision of prognosis in CRC patients, ultimately contributing to more personalized treatment strategies.

### 3.6 Correlation analysis between immune cell infiltration, immune microenvironment, tumor mutation burden, and risk scoring

**Figure 6:**
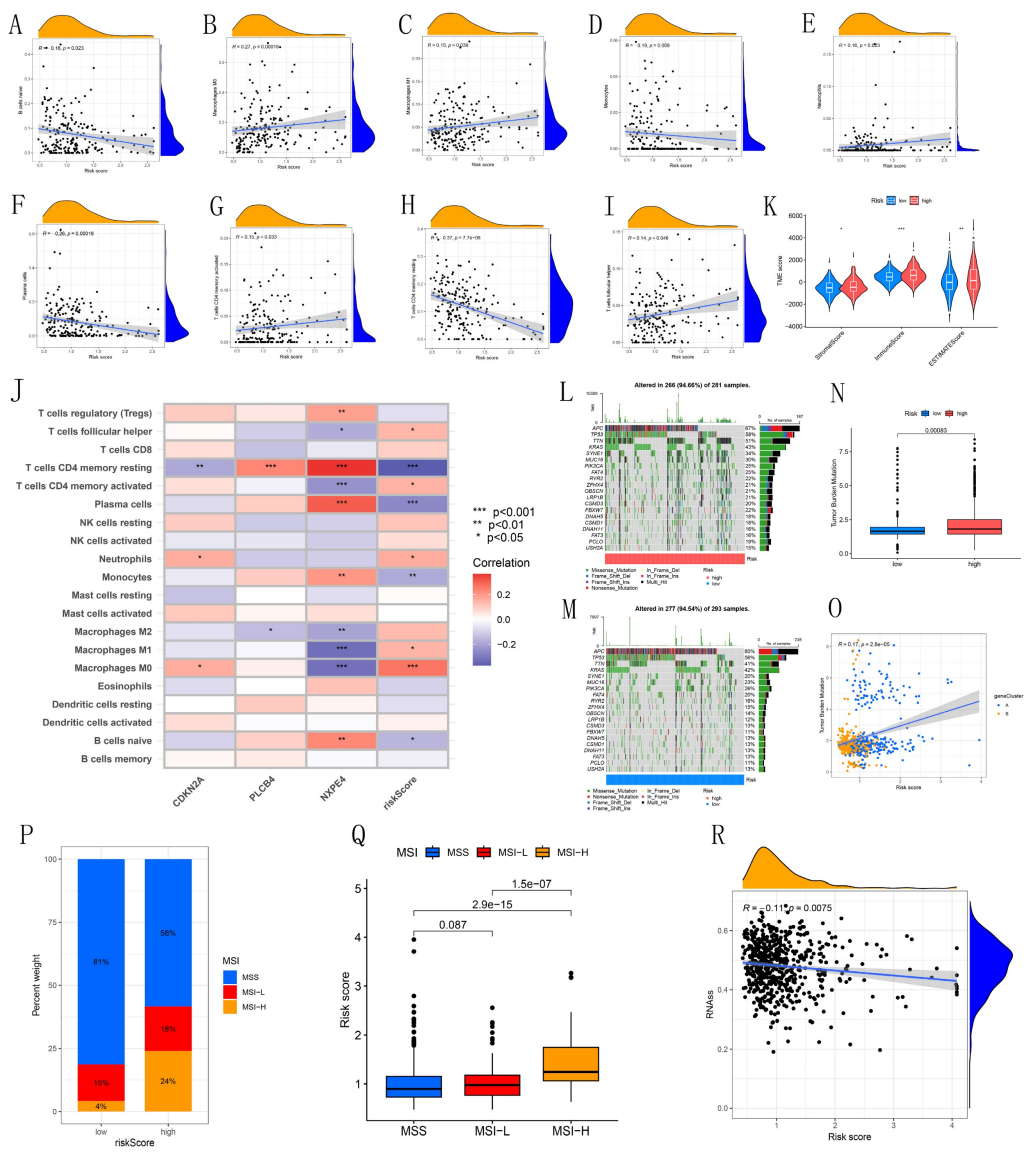
Immunological landscape analysis. (A-I) Correlation analysis between risk score and immune infiltrating cells. (J) Correlation between model-built genes and immune cells. (K) Violin maps for differential analysis of the tumor microenvironment. (L-M) Waterfall plots of tumor mutation burden for high- and low-risk groups. (N) Bar chart showing the difference in TMB between high- and low-risk groups. (O) Scatter plot of the correlation between TMB values and risk scores. (P) MSI characteristics of the diverse CPS-score subgroups. (Q) Differences in CPS score between MSS, MSI-L, and MSI-H. (R) Scatterplot of stem cell correlations.

Our study reveals associations between different immune cell types and risk scores. Specifically, T follicular helper cells, activated CD4 memory T cells, neutrophils, M1 macrophages, and M0 macrophages showed positive correlations with risk scores, while naive B cells, monocytes, plasma cells, and resting CD4 memory T cells displayed negative correlations (Figures 6A-I). CDKN2A, PLCB4, and NXPE4 were also significantly correlated with various immune cells (Figure 6J). Violin plots illustrated tumor microenvironment characteristics across high and low-risk cohorts, showing significantly higher StromalScore, ImmuneScore, and ESTIMATEScore in the high-risk group (Figure 6K). This finding suggests that the immune microenvironment may be more active in high-risk patients, potentially influencing tumor progression. Examination of the somatic mutation spectrum identified APC, TP53, and TTN as the top three genes with elevated mutation frequencies in both high and low-risk groups, providing insights into the relationship between genetic variability and tumor risk stratification (Figures 6L-M). Additionally, TMB calculations showed a notable positive correlation with risk scores, suggesting that elevated TMB may indicate higher tumor risk (Figures 6N-O). These correlations emphasize the importance of integrating immune and genetic factors for more accurate risk assessment in CRC patients, potentially guiding personalized treatment strategies.

### 3.7 Analysis of risk scores with microsatellite instability and stem cell correlation, and drug sensitivity between different risk groups

This study explored the association between microsatellite instability (MSI) and risk scores. In the low-risk cohort, the distribution of MSI statuses was 81% microsatellite stable (MSS), 15% MSI-Low (MSI-L), and 4% MSI-High (MSI-H). In contrast, the high-risk cohort exhibited a decrease in MSS to 58%, an increase in MSI-L to 18%, and a significant rise in MSI-H to 24% (Figure 6P). These results indicate a notable correlation between elevated risk scores and increased microsatellite instability, suggesting that higher risk is associated with a more unstable genomic environment. Further analysis of risk scores across different MSI statuses revealed that the median risk score was significantly higher in the MSI-H group compared to the MSS and MSI-L groups, highlighting a strong association between high microsatellite instability and increased risk scores (Figure 6Q). Additionally, the analysis of *RNA*-SS scores showed a negative correlation with risk scores (Figure 6R). Drug sensitivity analysis indicated that most chemotherapy drugs were more effective in the high-risk group (Figure S5).

### 3.8 Differential expression of CDKN2A, NXPE4, and PLCB4 in normal and tumor tissues of CRC

**Figure 7:**
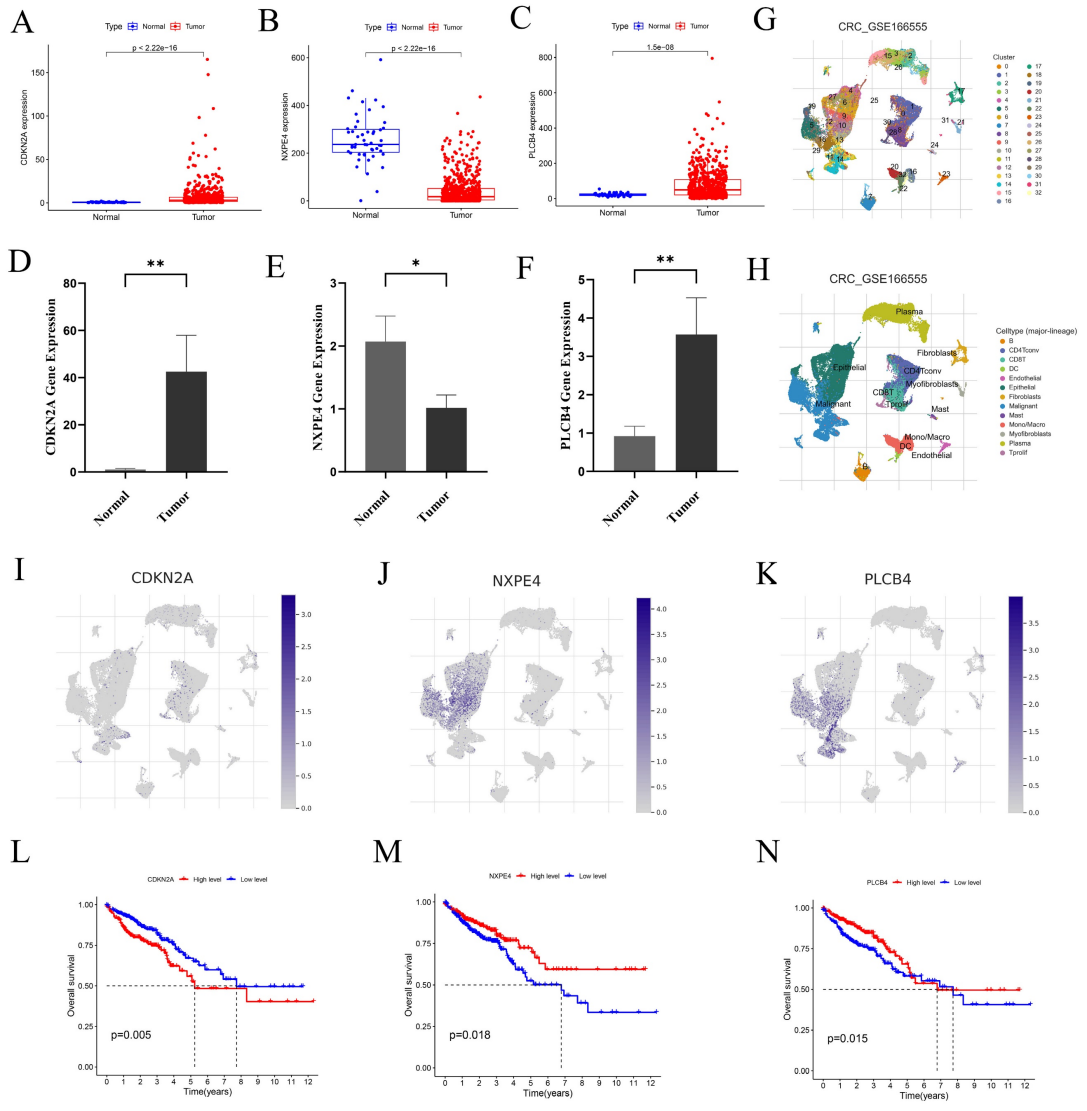
Three key genes exhibit expression differences at both the single-cell level and the transcriptome level. (A-C) Expression levels of CDKN2A, NXPE4, and PLCB4 in CRC tumor tissues compared to normal tissues from the TCGA database. Statistical significance was assessed using independent sample t-tests. (D-F) Validation of gene expression levels in CRC tumors and normal tissues via qRT-PCR. Expression differences were evaluated using paired t-tests, revealing statistically significant results. (G-H) UMAP plots from the GSE166555 dataset showing the distribution of CDKN2A, NXPE4, and PLCB4 expression at the single-cell level. These plots illustrate the heterogeneity of gene expression within individual CRC cells. (I-K) Detailed expression distribution maps for CDKN2A, NXPE4, and PLCB4 at the single-cell level, highlighting variability across different cell populations in CRC. Statistical analysis of single-cell data was performed using non-parametric tests, with significance thresholds set at *P<0.05, **P<0.01, and ***P<0.001.

We examined the expression levels of CDKN2A, NXPE4, and PLCB4 in normal and tumor tissues using data from the TCGA database (Figures 7A-C). To validate these findings, qRT-PCR was performed. Results showed increased levels of CDKN2A and PLCB4 in tumor tissues, whereas NXPE4 expression was higher in normal tissues (Figures 7D-F).

### 3.9 Differential expression of CDKN2A, NXPE4, and PLCB4 in scRNA-seq

To investigate the roles of CDKN2A, NXPE4, and PLCB4 at the single-cell level, we performed scRNA-seq analysis on the GSE166555 dataset using the TISCH database. Cell clustering and annotation identified 13 cell types (Figures 7G-H, Figure S6). CDKN2A was primarily expressed in proliferative T cells, CD8 T cells, and malignant cells, PLCB4 was mainly found in malignant and endothelial cells, and NXPE4 was predominantly present in epithelial cells (Figures 7I-K). These findings align with our qRT-PCR results. Kaplan-Meier survival analysis revealed that high CDKN2A expression is associated with poorer prognosis, while high PLCB4 and NXPE4 expressions are associated with better prognosis (Figures 7L-N). This highlights the potential of CDKN2A as a prognostic marker for aggressive disease, while PLCB4 and NXPE4 may act as favorable prognostic indicators, indicating their distinct functions in the progression of CRC and patient outcomes.

### 3.10 Differential Expression of CDKN2A in CRC

**Figure 8:**
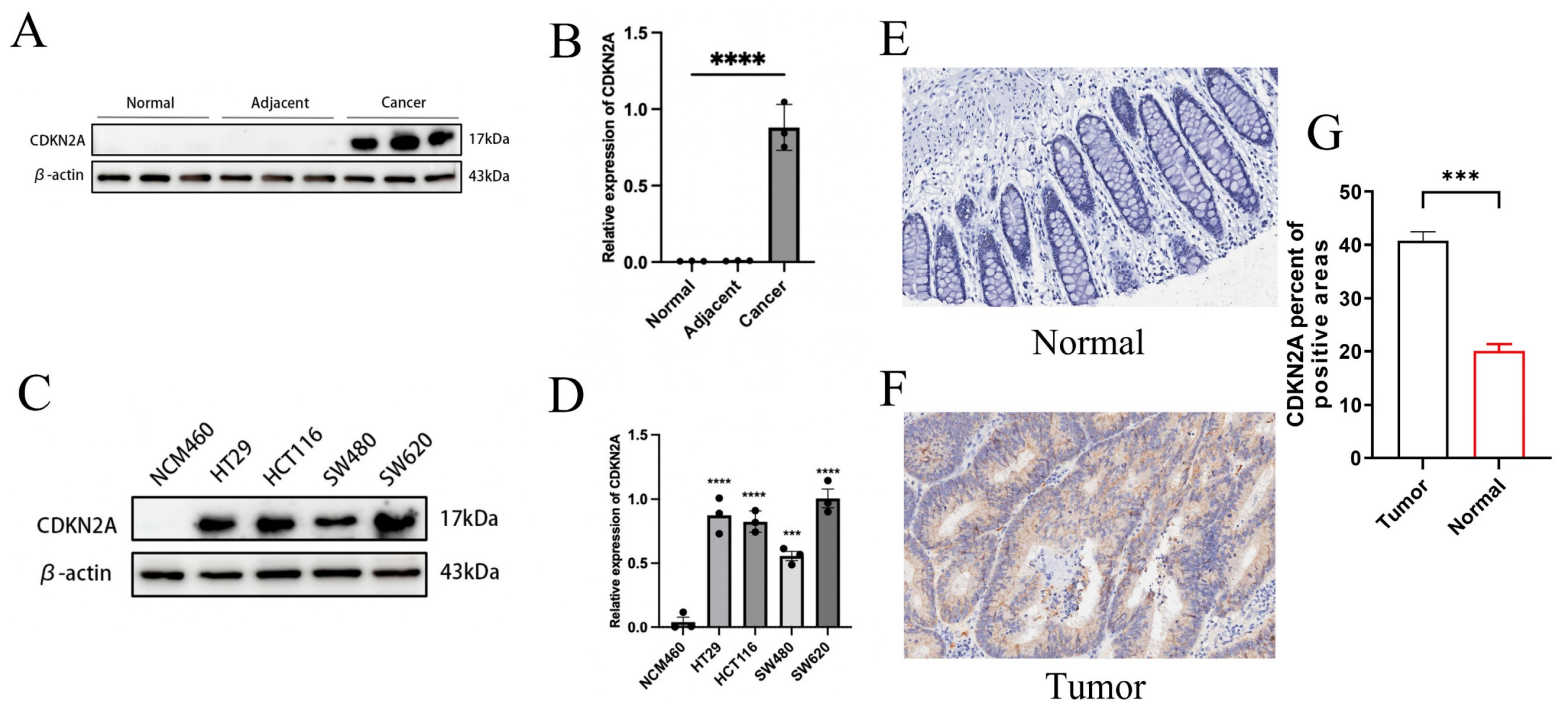
Differential expression of CDKN2A. (A-B) Expression of CDKN2A in normal, adjacent, and colon cancer tissues assessed by WB. Statistical significance between groups was determined using one-way ANOVA, with significance denoted by **** for *P* < 0.0001. (C-D) WB results showing CDKN2A expression in normal intestinal epithelial cells (NCM460) compared to multiple CRC cell lines (HT29, HCT116, SW620, SW480). Statistical significance was calculated by one-way ANOVA, with symbols indicating **P < 0.01, ***P < 0.001, and ****P < 0.0001. (E-F) Representative images from our IHC staining of CDKN2A in normal and colon cancer tissues, illustrating protein localization and abundance. (G) Quantitative analysis of CDKN2A staining intensity in colon cancer versus normal tissues, with statistical significance assessed by independent samples t-tests, indicated by *** for *P* < 0.001.

We analyzed CDKN2A expression across various tissue samples, including normal, adjacent, and tumor tissues, as well as normal intestinal epithelial cells (NCM460) and multiple CRC cell lines (HT29, HCT116, SW620, SW480). Elevated protein levels of CDKN2A were observed in both cancerous tissues and tumor cell lines (Figures 8A-D). IHC analysis showed that CDKN2A is primarily expressed in small amounts in the nucleus in normal tissues, while in Colon cancer tissue, its expression is significantly localized to the cytoplasm, with a small amount still present in the nucleus (Figures 8E-F). Further quantitative assessment corroborated this finding, indicating that the proportion of CDKN2A positive expression in cancer tissues was substantially elevated relative to normal tissues (Figure 8G). These findings suggest a pronounced expression trend of CDKN2A in CRC tissues, indicating its potential significance in the initiation and advancement of CRC.

## 4. Discussion

Recent research has highlighted the significant role of copper ions in cancer progression, particularly their impact on tumor proliferation, movement, and vascular development 23. Copper ions influence cancer cell survival and death by affecting mitochondrial functions and copper transport across the extracellular matrix 24. This has led to new therapeutic strategies, such as targeting cuproptosis—a novel form of cell death that disrupts mitochondrial acylated proteins and Fe-S cluster proteins 25. The dysregulation of CRGs is closely linked to CRC progression and prognosis, positioning these genes as potential therapeutic targets 26. Our study investigates the variation in CRG copy numbers in CRC, exploring their relationships with clinical features, the tumor microenvironment, and immune cell infiltration at both transcriptome and single-cell levels. We identified CDKN2A, NXPE4, and PLCB4 as key players in cuproptosis and potential targets for CRC therapy.

Chromosomal copy number variations are crucial in tumor development, particularly in CRC 27, 28. Research by Zhang *et al.* has shown that chromosomal instability and copy number changes significantly affect tumor cell genetic heterogeneity and adaptability 29. For instance, HER2 gene amplification in breast cancer leads to overproduction of the HER2 protein, which correlates with aggressive tumor behavior and poor prognosis 30. Similarly, TP53 deletions are common across various cancers, leading to the loss of tumor suppressor functions and promoting tumor growth and resistance to apoptosis 31. Our study integrates CNV and gene expression analyses, revealing that DBT has the highest mutation rate among the 19 CRGs studied, mainly due to deletions on chromosome 1. DBT expression is higher in normal colorectal tissues compared to cancerous ones, suggesting that DBT may be crucial for maintaining normal colorectal tissue function, and its copy number alterations could contribute to cancer progression.

Additionally, we developed a prognostic risk model based on differentially expressed CRGs in CRC. This model, incorporating CDKN2A, PLCB4, and NXPE4, serves as an independent prognostic marker, offering a comprehensive assessment of CRC prognosis beyond traditional TNM staging. It enhances the precision and dynamics of tumor progression and prognosis monitoring. Similar models have been developed in previous studies; for example, Liang *et al.* used cancer stem cell-associated genes to establish a CRC risk model 32, while Shi *et al.* investigated CRGs in hepatocellular carcinoma (HCC) and identified eight CRGs linked to HCC prognosis. Their models predicted 1-, 3-, and 5-year survival rates with AUC values of 0.658, 0.647, and 0.629, respectively 33. Our model, using CDKN2A, PLCB4, and NXPE4, demonstrated improved stability with AUC values ranging from 0.634 to 0.688 for 1 year, 0.600 to 0.640 for 3 years, and 0.562 to 0.637 for 5 years. This model offers a cost-effective and clinically viable alternative to whole transcriptome sequencing, making it a valuable tool for CRC diagnostics and therapeutics.

Recent studies underscore the critical role of immune cell infiltration, particularly tumor-infiltrating lymphocytes (TILs), in modulating the tumor microenvironment 34, 35. Our research reveals that high-risk CRC patients exhibit increased infiltration of M0 macrophages, which are typically associated with adverse outcomes. In contrast, low-risk patients show higher levels of resting memory CD4+ T cells and plasma cells. Resting memory CD4+ T cells are essential for regulating tumor dynamics and maintaining long-term immune memory through specific regulatory mechanisms 36. These cells remain dormant until activated by tumor antigens, enabling rapid immune responses that are crucial for preventing tumor recurrence 37. Jakic *et al.* report that these memory cells enhance immune responses by releasing cytokines such as IL-2 and IFN-γ, which boost the cytotoxic activities of other immune cells, including CD8+ T cells 38. This enhancement improves immune surveillance and combats tumor proliferation. Additionally, resting memory CD4+ T cells play a key role in monitoring long-standing tumors or minimal residual disease, providing ongoing protection and aiding in the prevention of tumor reemergence 39. Conversely, M0 macrophages promote tumor progression and growth through various mechanisms 40. They secrete significant amounts of pro-inflammatory cytokines and growth factors, such as TNF-α and VEGF, which support tumor cell growth, survival, and angiogenesis by providing essential nutrients and oxygen. M0 macrophages also release immunosuppressive cytokines like IL-10 and TGF-β, which can diminish T cell activity and impair the immune system's ability to target tumor cells, thereby facilitating tumor evasion 41. Furthermore, M0 macrophages contribute to tumor invasiveness and metastasis by releasing matrix metalloproteinases (MMPs) that degrade the extracellular matrix, promoting tumor cell migration 42. They also induce resistance to chemotherapy and radiotherapy by activating pathways such as NF-κB, STAT3, and PI3K/Akt 43. Our findings highlight how specific immune cells respond to and influence disease progression across different risk levels, offering valuable insights into their roles in immune-related diseases.

We identified three key genes—CDKN2A, PLCB4, and NXPE4—that exhibited significant differences at both the transcriptomic and single-cell levels. Survival analysis indicates that these genes are closely related to CRC prognosis. PLCB4, a crucial signal transduction enzyme, regulates cell proliferation, differentiation, and survival by modulating IP3 and DAG levels, affecting calcium signaling and the protein kinase C pathway 44. High expression of PLCB4 in tumor tissues, malignant cells, and endothelial cells correlates with its role in promoting tumor proliferation and angiogenesis. Typically, such expression patterns are associated with aggressive tumors and poor prognosis 45. However, our survival analysis shows that high PLCB4 expression is linked to better prognosis in CRC patients. This suggests that PLCB4's role in tumor biology may be more complex, potentially enhancing angiogenesis and improving therapeutic drug delivery, which could increase treatment efficacy 46. Additionally, PLCB4's role in regulating calcium signaling might help cells resist cuproptosis, a form of cell death induced by copper-based treatments 47. This resistance could reduce stress-induced cell death in other therapeutic scenarios, thereby positively influencing prognosis 48. In contrast, NXPE4 expression is generally lower in CRC tissues and cells compared to normal tissues, suggesting that NXPE4 may be important for maintaining normal cell functions and inhibiting tumor progression. Survival analysis indicates that low NXPE4 expression is associated with poorer prognosis, highlighting its potential role in tumor suppression. Reduced NXPE4 expression may affect CRC cell sensitivity to cuproptosis, as cuproptosis depends on cellular metabolic states and disturbances in fatty acid metabolism 49. Lower NXPE4 functionality could make tumor cells more susceptible to copper ion toxicity, exacerbating cuproptosis 50. In summary, PLCB4 and NXPE4 exhibit distinct expression patterns and functions across various CRC tissue and cell types, reflecting their complex and dual roles in tumor biology. Understanding these genes' roles is crucial for developing targeted therapies and predicting treatment outcomes. Future research should further explore these molecules' specific interactions in the TME and their impact on treatment responses, providing more precise and effective options for CRC patients.

This study primarily investigates the differential expression of CDKN2A protein across various tissues and cell types in CRC. Our results reveal that CDKN2A is significantly more expressed in tumor tissues compared to normal and adjacent non-tumor tissues. Additionally, CDKN2A expression levels are markedly higher in six CRC cell lines than in normal intestinal epithelial cells. Notably, low CDKN2A expression correlates with better prognosis in CRC patients. CDKN2A functions as a tumor suppressor gene, with its encoded protein, p16INK4a, inhibiting CDK4/6 and blocking the transition from G1 to S phase of the cell cycle 51. This action is crucial for cell cycle regulation. CDKN2A also stabilizes the p53 protein through its p14ARF subunit, which enhances the cellular response to DNA damage, promotes apoptosis, and induces senescence 52. Shi *et al.* have suggested that CDKN2A might resist cuproptosis by regulating glycolysis and copper ion homeostasis. This mechanism could be associated with malignant phenotypes and changes in the tumor microenvironment, offering new insights into therapeutic strategies for CDKN2A-expressing CRC 53. Our observations show that CDKN2A is highly expressed in malignant CRC cells, potentially enhancing cellular resistance to oxidative stress. This upregulation of antioxidant enzymes, such as superoxide dismutase and glutathione peroxidase, may help cells mitigate oxidative damage caused by copper ions, thereby reducing the risk of copper-induced cell death 54. High CDKN2A expression might also promote mitochondrial function, improving energy production efficiency and helping cells manage the increased energy demands triggered by copper ions 55. By maintaining energy balance and mitochondrial health, CDKN2A helps cells resist the death pressures caused by copper ions 56. Furthermore, CDKN2A's role in regulating the cell cycle under copper-induced stress conditions contributes to cellular stability, influencing survival or death decisions 57. This is crucial for tumor cell survival, potentially enhancing resistance to chemotherapeutic drugs 58. Therefore, the expression patterns and functional complexity of CDKN2A underscore its importance as a prognostic marker and potential therapeutic target in CRC. Further exploration of CDKN2A's role in cuproptosis regulation could lead to more effective treatment strategies.

However, our study has several limitations. Primarily, our data is derived from the TCGA and GEO databases and lacks external validation. Additionally, the verification of expression profiles has been limited to tissue and cell levels. To enhance our understanding of key genes in CRC, further assays of cellular functions and the development of animal models in a physiological environment are necessary.

## Supplementary Material

Supplementary figures and table.
