# Supplementary material for: Single-cell and Multi-omics Analysis Confirmed the Signature and Potential Targets of Cuproptosis in Colorectal Cancer
Source: J Cancer. 2025 Jan 13;16(4):1264–80. doi: 10.7150/jca.104702 (PMC11786029; doi:10.7150/jca.104702)
Supplement: Supplementary file 1 — Supplementary figures and table. [file jcav16p1264s1.pdf]

Supplementary Material

Supplementary Figure 1. Clustering analysis of cuproptosis-associated genes.

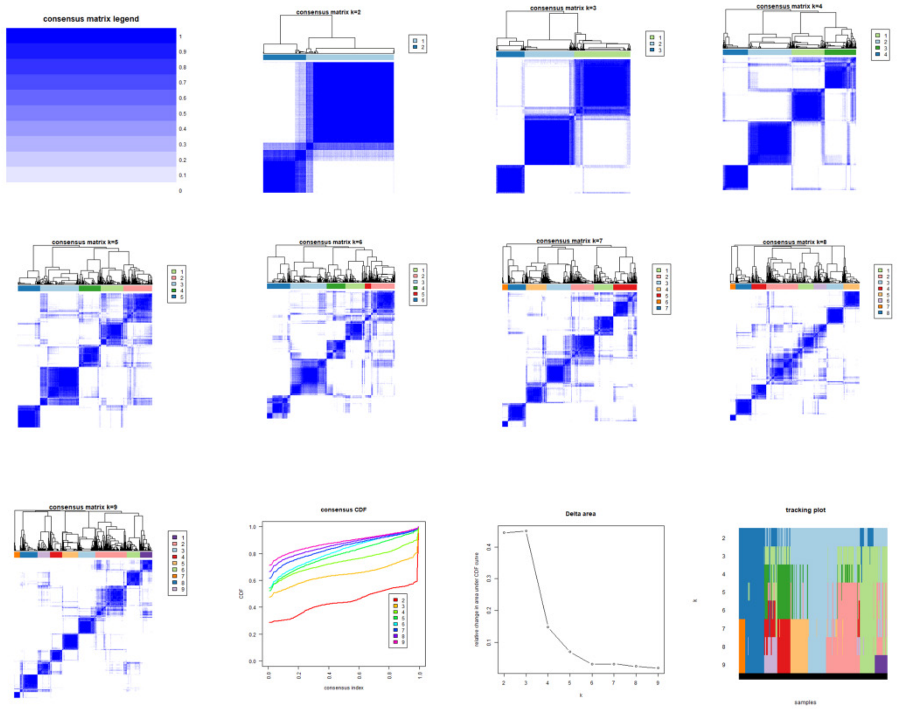

Supplementary Figure 2. Venn diagram of significantly differentially expressed genes in cuproptosis typing.

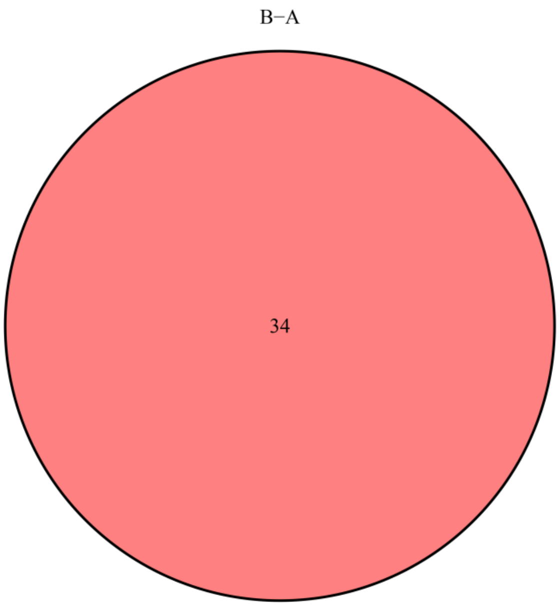

Supplementary Figure 3. Clustering analysis of differentially expressed genes.

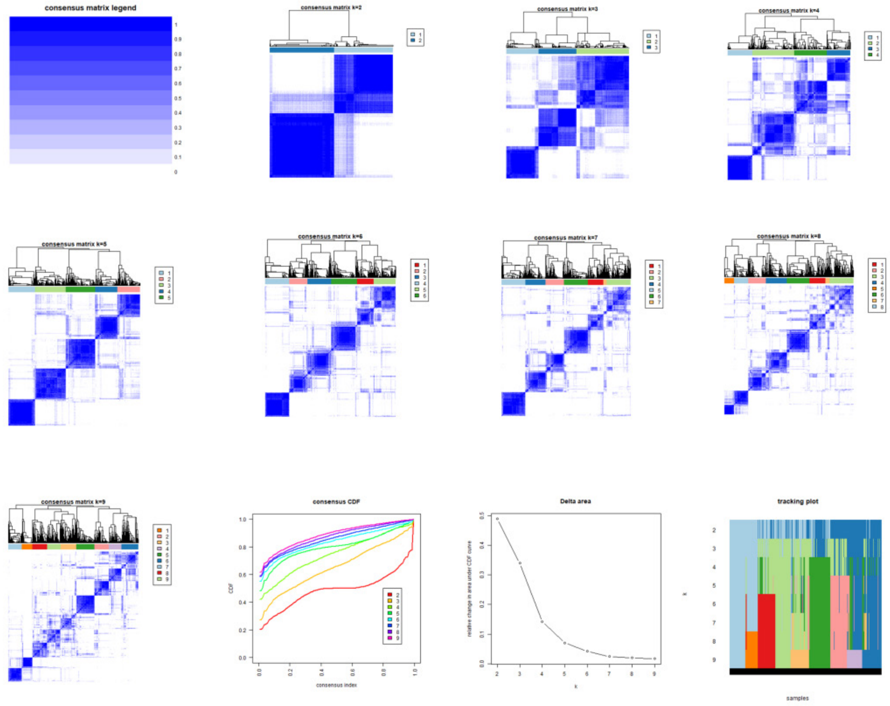

Supplementary Figure 4. Changes in the concordance index for survival prediction between risk models and different clinical factors.

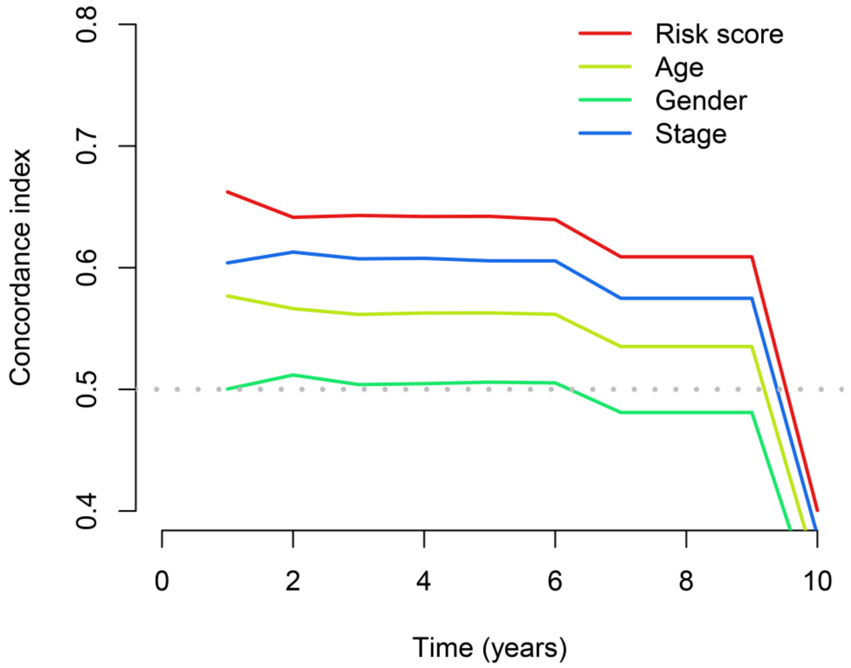

Supplementary Figure 5. Box plot of drug sensitivity analysis between different risk groups.

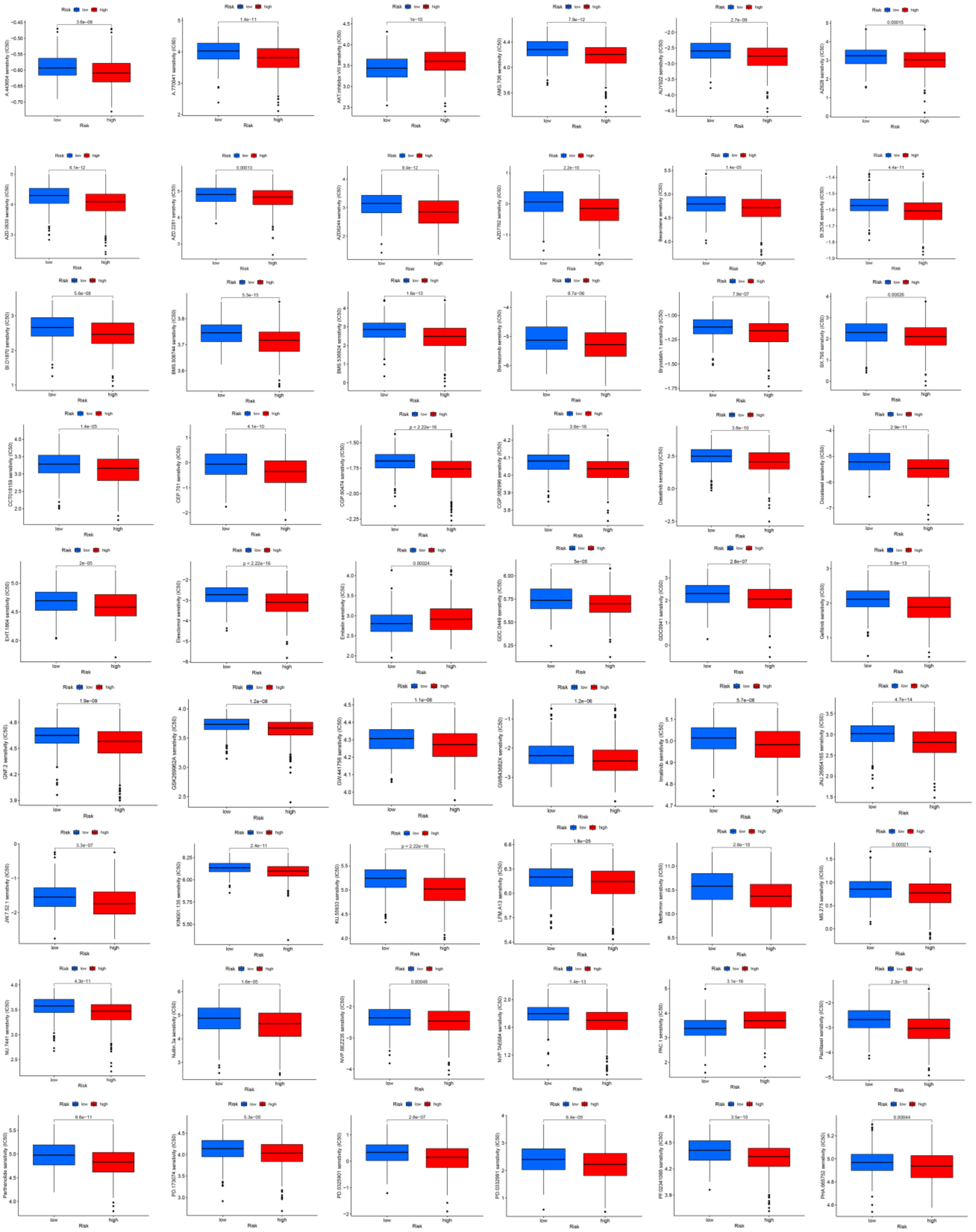

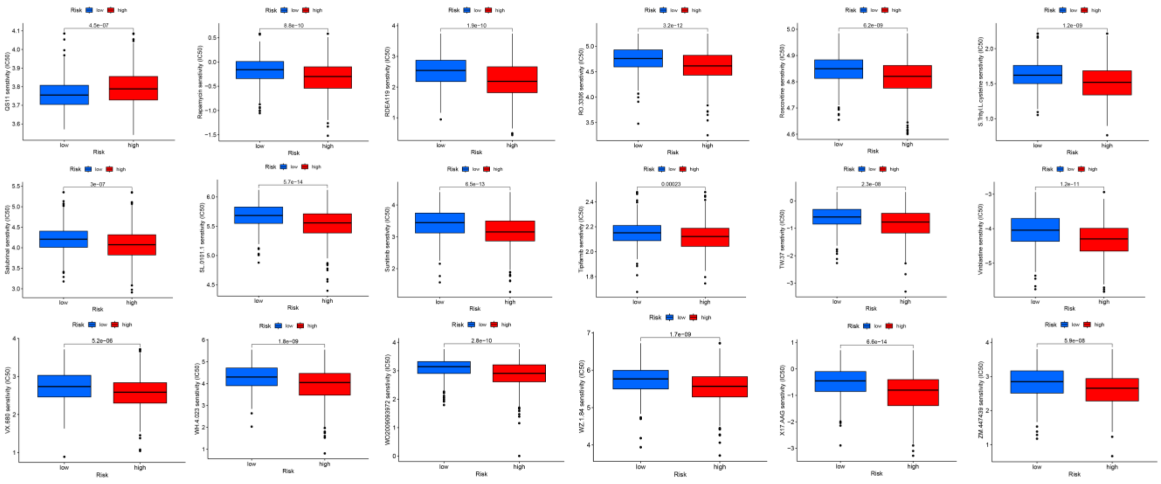

Supplementary Figure 6. A-B Proportions of different cell types in single-cell analysis. C Violin plot of single-cell expression of hub genes.

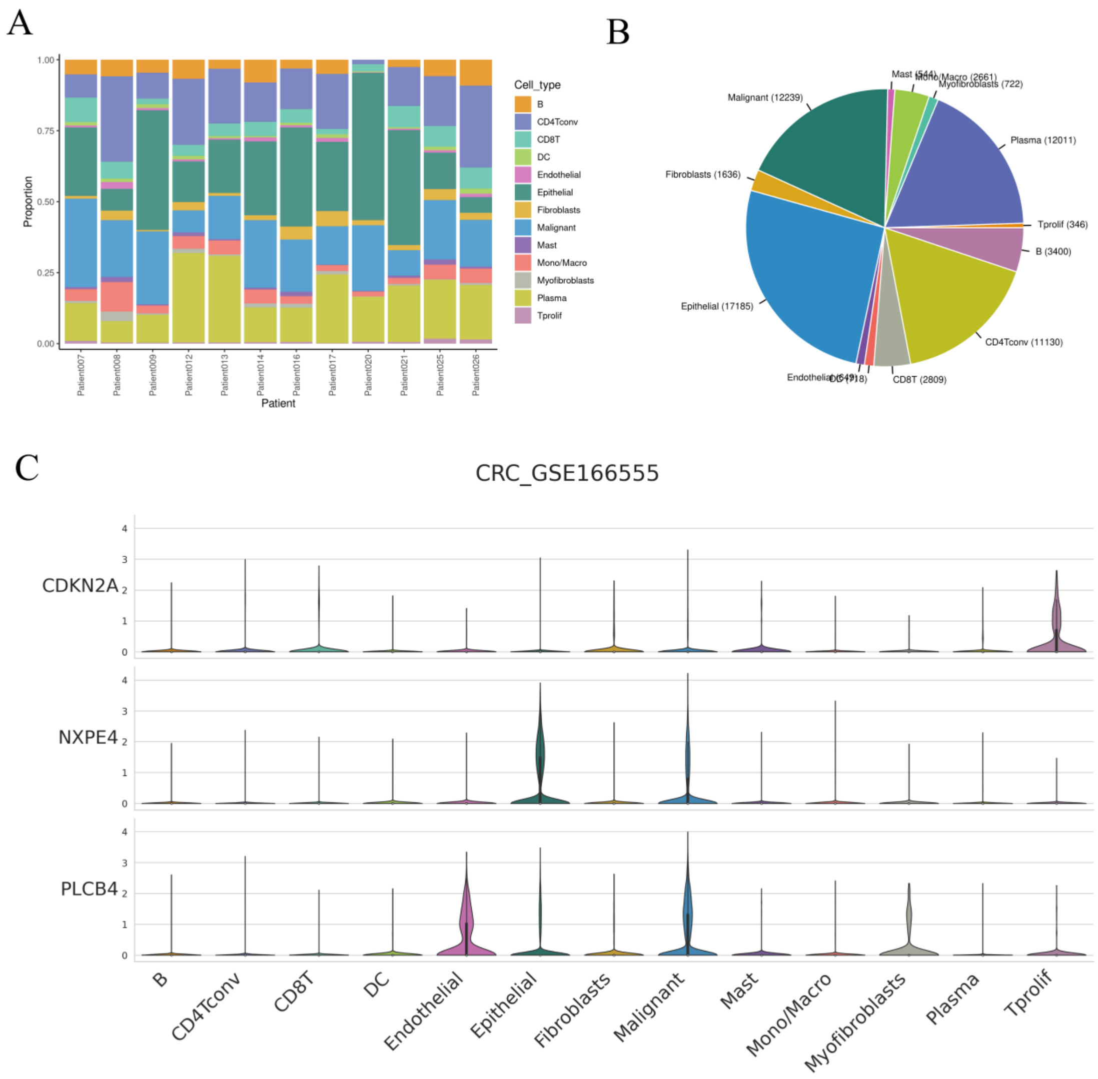

Supplementary Table 1. Primers

Gene symbol	Primer sequence
CDKN2A	F:CGATGTCGCACGGTACCTG<br>R:GACCTTCCGCGGCATCTATG
PLCB4	F:GCATGATGGAAGCTGGAGAA<br>R:CTTCCTCCTCTTGGTCAGCA
NXPE4	F:AGATCCCACCCAGACCTTTC<br>R:CCTGCAGTACGTATCTCGAG
